# Probabilistic Motor Sequence Yields Greater Offline and Less Online Learning than Fixed Sequence

**DOI:** 10.3389/fnhum.2016.00087

**Published:** 2016-03-02

**Authors:** Yue Du, Shikha Prashad, Ilana Schoenbrun, Jane E. Clark

**Affiliations:** ^1^Department of Kinesiology, University of MarylandCollege Park, MD, USA; ^2^Applied Mathematics and Statistics, and Scientific Computation Program, University of MarylandCollege Park, MD, USA; ^3^Neuroscience and Cognitive Science Program, University of MarylandCollege Park, MD, USA

**Keywords:** online learning, offline learning, probabilistic sequence, sequence knowledge, procedural memory, declarative memory, fast motor sequence learning

## Abstract

It is well acknowledged that motor sequences can be learned quickly through online learning. Subsequently, the initial acquisition of a motor sequence is boosted or consolidated by offline learning. However, little is known whether offline learning can drive the fast learning of motor sequences (i.e., initial sequence learning in the first training session). To examine offline learning in the fast learning stage, we asked four groups of young adults to perform the serial reaction time (SRT) task with either a fixed or probabilistic sequence and with or without preliminary knowledge (PK) of the presence of a sequence. The sequence and PK were manipulated to emphasize either procedural (probabilistic sequence; no preliminary knowledge (NPK)) or declarative (fixed sequence; with PK) memory that were found to either facilitate or inhibit offline learning. In the SRT task, there were six learning blocks with a 2 min break between each consecutive block. Throughout the session, stimuli followed the same fixed or probabilistic pattern except in Block 5, in which stimuli appeared in a random order. We found that PK facilitated the learning of a fixed sequence, but not a probabilistic sequence. In addition to overall learning measured by the mean reaction time (RT), we examined the progressive changes in RT within and between blocks (i.e., online and offline learning, respectively). It was found that the two groups who performed the fixed sequence, regardless of PK, showed greater online learning than the other two groups who performed the probabilistic sequence. The groups who performed the probabilistic sequence, regardless of PK, did not display online learning, as indicated by a decline in performance within the learning blocks. However, they did demonstrate remarkably greater offline improvement in RT, which suggests that they are learning the probabilistic sequence offline. These results suggest that in the SRT task, the fast acquisition of a motor sequence is driven by concurrent online and offline learning. In addition, as the acquisition of a probabilistic sequence requires greater procedural memory compared to the acquisition of a fixed sequence, our results suggest that offline learning is more likely to take place in a procedural sequence learning task.

## Introduction

In the laboratory, studies employing the serial reaction time (SRT) task (Nissen and Bullemer, 1987) have demonstrated that adults can learn a motor sequence quickly within a single training session (i.e., in 4 to 8 practice blocks; Nissen and Bullemer, 1987; Willingham et al., 1989; Robertson, 2007). This initial stage of motor sequence learning is referred to as fast learning that leads to the initial acquisition of sequences (Honda et al., 1998; Karni et al., 1998; Walker et al., 2002; Dayan and Cohen, 2011; Censor et al., 2012). Fast learning develops over the course of a single training session, where an individual practices a new motor sequence and demonstrates considerable performance improvement. It has been suggested that such improvement in the performance of motor sequences are driven by online learning (Bornstein and Daw, 2012, 2013; Verstynen et al., 2012), where performance progressively improves as the task is practiced. After the fast learning stage, performance is strengthened without further practice (i.e., offline learning) by an early offline boost (Hotermans et al., 2006; Schmitz et al., 2009) or memory consolidation (Robertson et al., 2004b, 2005; Brown and Robertson, 2007a; Nettersheim et al., 2015). To date, it is unclear whether offline learning drives the acquisition of motor sequence in the fast learning stage. The purpose of this study, therefore, is to examine whether fast learning of a motor sequence arises from offline learning. Furthermore, given that offline learning in the SRT task has been found to be associated with procedural memory (Robertson et al., 2004a; Brown and Robertson, 2007a,b), we further investigate whether a bias towards procedural or declarative memory in the SRT task modulates offline and online sequence learning.

Learning motor sequences in the SRT tasks typically involves both procedural and declarative memory (Willingham et al., 1989; Curran and Keele, 1993; Reber and Squire, 1994; Willingham and Goedert-Eschmann, 1999; Destrebecqz and Cleeremans, 2001; Brown and Robertson, 2007a; Robertson, 2007). In this task, participants press keys on the keyboard to respond to sequential visual stimuli that are presented in a pattern (e.g., a fixed order). Since participants are not informed of the presence of the sequence, learning in the SRT task requires procedural memory. However, participants may recognize the presence of the sequence after they perform the task and thus form a declarative memory of the sequence (Perruchet et al., 1997; Willingham and Goedert-Eschmann, 1999). This entanglement of procedural and declarative learning suggests the infeasibility of eliminating or isolating either of them from the SRT task. Nonetheless, manipulating the sequence type and the preliminary knowledge (PK) of the sequence can modulate procedural or declarative learning. Particularly, it has been shown that learning a probabilistic sequence favors more procedural memory compared to learning a fixed sequence (Jiménez et al., 1996; Song et al., 2007). In contrast, PK of the sequence facilitates declarative learning (Curran and Keele, 1993; Curran, 1997; Destrebecqz, 2004).

In this study, we bias the involvement of procedural/declarative memory by manipulating the sequence type and PK of the sequence in the SRT task to examine whether offline or online learning mediate the acquisition of motor sequences in the fast learning stage. Before the experiment, we informed half of the participants that the visual stimuli followed a specific pattern, but no further information was provided about the sequence. No information about the presence of a sequence was provided to the other participants. The participants were further divided into two groups. In one group, the visual stimuli followed a fixed sequence (i.e., 10 repetitions of a 12-trial sequence) while in the other group; the visual stimuli followed a probabilistic sequence that was generated by a first-order Markov process. We found that a motor sequence is learned quickly through concurrent online and offline learning. However, the involvement of procedural or declarative memory mediated the use of online and offline learning. Particularly, learning of a fixed sequence arose from greater online learning. In contrast, acquisition of a probabilistic sequence resulted from significant offline learning, regardless of PK. These results suggest that the involvement of procedural and declarative memory modulates how a motor sequence is learned in the fast learning stage.

## Materials and Methods

This study was carried out in accordance with the recommendations and approval of the Institutional Review Board at the University of Maryland, College Park. All participants signed consent forms prior to their participation. Each participant received $10 after the completion of the experiment.

### Participants

Forty-eight right-handed adults (24 males, see Table 1) were randomly assigned to one of four groups: fixed sequence with PK of the sequence (PK_Fixed; mean age: 21.8 ± 1.91), fixed sequence without PK of the sequence (NPK_Fixed; mean age: 21.5 ± 1.41), probabilistic sequence with PK of the sequence (PK_Prob; mean age: 21.2 ± 0.893), and probabilistic sequence without PK of the sequence (NPK_Prob; mean age; 21.3 ± 0.830). All participants completed a health questionnaire to exclude those with any neurological and motor impairments, the Edinburgh Handedness Inventory (Oldfield, 1971) to assess that participants were right-handed, and the Global Physical Activity Questionnaire (Armstrong and Bull, 2006) to insure that groups did not differ in their level of physical activity.

**Table 1:** **Participant demographic information**.

Group	Age (years)^#^	Sex
PK_Fixed	21.8 ± 1.91	6 females; 6 males
NPK_Fixed	21.5 ± 1.41	6 females; 6 males
PK_Prob	21.2 ± 0.893	6 females; 6 males
NPK_Prob	21.3 ± 0.830	6 females; 6 males

*^#^There were no significant differences between the groups in age, F_(3,47)_ = 0.564, p = 0.642*.

### Serial Reaction Time Task

**Figure 1:**
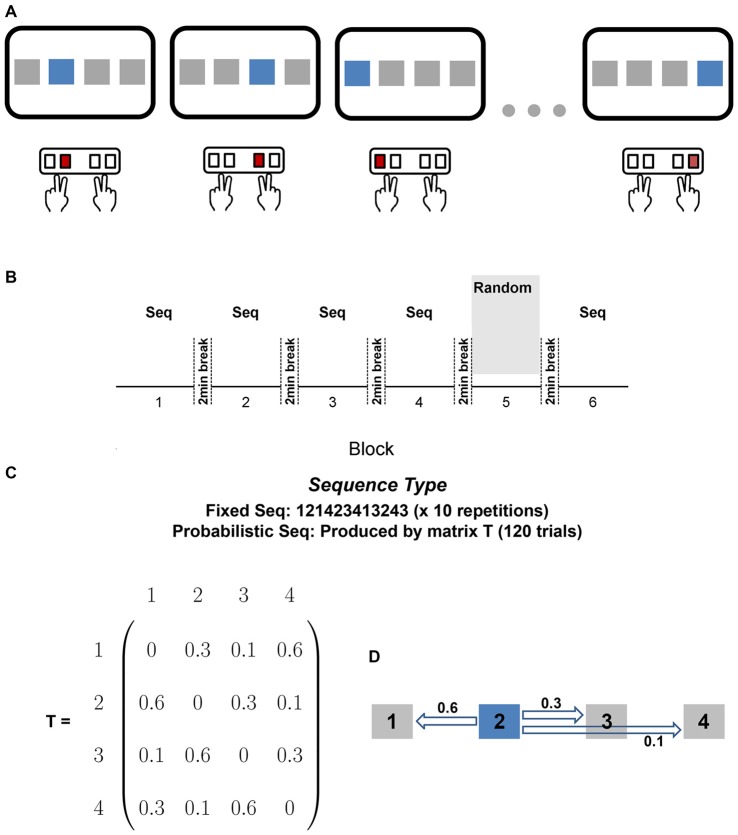
**(A)** Experimental setup. At the beginning of each trial, a stimulus appeared in one of four squares on the screen and the participant pressed the key that corresponded to the location of the stimulus. Participants placed the middle finger of their left hand on the keyboard’s “D” key, the index finger of their left hand on the “F” key, the index finger of their right hand on the “J” key, and the middle finger of their right hand on the “K” key. **(B)** Experimental paradigm. Participants performed the learning blocks (blocks 1–4) with either the fixed or probabilistic sequence, followed by randomly ordered stimuli in block 5, and ended with the same sequence in block 6. All blocks consisted of 120 trials. Participants were given a mandatory 2 min break between each block. **(C)** Sequence Types. Participants were randomly assigned to the fixed sequence group or the probabilistic sequence group. The probabilistic sequence was created using the probabilities defined in the transitional matrix, T. **(D)** Example of how the probabilistic sequence was created using matrix T. If the current stimulus is 2, there is a probability of 0.6 that the next stimulus will be 1, a 0.3 probability that the next stimulus will be 3, and a 0.1 probability that the next stimulus will be 4.

Participants were seated in front of a computer monitor (19″) and keyboard. Participants placed the middle finger of their left hand on the keyboard’s “D” key, the index finger of their left hand on the “F” key, the index finger of their right hand on the “J” key, and the middle finger of their right hand on the “K” key (see Figure 1A). At the beginning of each trial, a mouse appeared in one of four squares on the screen and the participant pressed the key that corresponded to the location of the stimulus. After the participant pressed a key, the next stimulus appeared after an interval of 300 ms. No visual feedback was provided to participants and a wooden board blocked vision of their finger position. Participants were first randomly assigned to either the PK group or no preliminary knowledge (NPK) group and were further randomly assigned to either the fixed or probabilistic sequence. The probabilistic sequence was created based on a Markov chain transitional matrix with probabilities associated with each stimulus (Figures 1C,D). The probabilistic sequence was constrained such that the same stimuli were not repeated one after the other and that each stimulus appeared an equal number of times in each block.

There were a total of six blocks for all groups (see Figure 1B), each consisting of 120 trials. Prior to the first block, participants practiced a random sequence. These initial trials were included to ensure that participants were able to accurately associate each finger with a corresponding key before the experimental practice blocks commenced. That is, we observed that participants did not produce reaction times (RTs: amount of time taken to press the corresponding button after the stimulus was presented) that were slower than 2000 m because of incorrect key pressing. After the practice block, participants in the PK groups were informed that a sequence would be present in the subsequent blocks and that they should look for the sequence. No other information about the nature of the sequence was provided. The first four blocks (Blocks 1–4) were the learning blocks consisting of the 120-trial probabilistic sequence or the fixed sequence in which the sequence was repeated 10 times in each block. Block 5 consisted of 120 trials of stimuli occurring in a random order and block 6 consisted of the assigned probabilistic or fixed sequence (Figures 1C,D). Participants were given a 2 min mandatory break between each block. The participants’ RT was recorded for each trial.

All participants completed a posttest after the completion of the six blocks to determine the amount of declarative knowledge of the sequence. Participants were first asked to recall the sequence and attempted to write down the 12 items of the sequence and rated how confident they were that the sequence they wrote was correct. Participants were then asked to complete a recognition task. They were given eight chunks (i.e., four three-element and four four-element chunks where two of each were correct) and were asked to choose the chunks they thought were included in the sequence.

### Data Analysis

The RTs were trimmed according to the individual participant’s mean and standard deviation. Within each block for an individual participant, any RT greater or less than 2.5 standard deviations was excluded from the analysis (Ratcliff, 1993; Whelan, 2008). Mean RTs were calculated for each block and were averaged across participants in each group. Learning was measured through a decrease in RT from block 1 to 4 (stimuli in assigned sequence) and an increase in RT from block 4 (stimuli in assigned sequence) to block 5 (stimuli in random order). Online learning was defined as the amount of learning within a block and was determined by performing a linear regression on the 120 RTs within a block. Offline learning was computed as the RT change after a short break without performing the task. Given that the fixed sequence consisted of 10 repetitions of a 12-item long sequence, the difference between mean RT of the last 12 taps in one block and that of the first 12 taps in the succeeding block was used to quantify offline learning. In addition, since participants typically acquire the sequence transitions of higher probabilities in probabilistic sequence learning (Hunt and Aslin, 2001; Howard et al., 2004; Bornstein and Daw, 2012), we expect that participants in the two probabilistic sequence groups would only learn sequential stimuli that were associated with transitional probabilities of 0.3 and 0.6 and fail to learn those associated with transitional probability of 0.1. Thus, we computed mean RT, offline- and online-learning of stimuli with transitional probabilities of 0.3 and 0.6 in the two probabilistic sequence groups.

A controversy regarding offline improvement in RT is whether the improvement results from reactive inhibition/fatigue (Rickard et al., 2008; Brawn et al., 2010) or it is driven by active learning mechanisms (i.e., offline learning; Eysenck and Frith, 1977; Robertson et al., 2004a). According to Eysenck and Frith (1977), in the case of reactive inhibition/fatigue-induced offline improvement, post-rest performance should return to the starting performance level before the rest or so called pre-rest performance, but without improvement over that level. In contrast, post-rest performance is superior to the pre-rest performance if offline improvement arises from offline learning. Given that RT increased (i.e., became slower) within blocks in some participants so that the mean RT of the last 12 taps may not reflect the pre-rest performance, we calculated corrected offline learning. Specifically, if RT increased (i.e., became slower) within the previous block, corrected offline learning was calculated by subtracting the amount of RT deterioration (i.e., negative online learning) within the previous block from the amount of offline learning so that the corrected offline learning reflects the difference between the pre-rest and post-rest performance. If RT improved (i.e., became faster) within the previous block, indicating no RT deterioration, corrected offline learning was the same as offline learning, computed as the difference between mean RT of the last 12 taps in the block and that of the first 12 taps in the succeeding block. We expect that all groups should exhibit the same amount of corrected offline learning (none), if offline improvement in RT observed in this study were caused by reactive inhibition or fatigue.

To measure the amount of declarative knowledge of the sequence, we calculated the recognition score as the number of correct chunks that participants chose in the recognition task. The recognition score was normalized by four as there were four correct chunks. To compare the recall score among participants, we calculated the number of three-element chunks that participants could recall. Given there were 12 three-element chunks in the fixed sequence, the number that a participant recalled was normalized by 12 to compute a percentage. To make the amount of declarative knowledge between probabilistic and fixed sequences comparable, the number of three-element chunks that participants could recall was also used in the two groups who performed the probabilistic sequence. Since participants only learned the stimulus transition with transitional probabilities of 0.3 and 0.6 (for details, see “Results” Section), there were 16 three-element chunks in the probabilistic sequence. Thus, the percent of recalled chunks was normalized by 16 in the two probabilistic sequence groups. Importantly, the chance level for guessing differed between the fixed and probabilistic sequence. Specifically, the chance level for a three-element chunk in the fixed sequence was 18.75% (i.e., given the first element, 75% chance for the second element and 25% chance for the third element) while it was 25% for a three-element chunk in the probabilistic sequence (i.e., given the first element, 50% chance for the second and third elements), we corrected the percentage of recalled chunks by the chance level specific to each sequence group.

### Statistical Analysis

A three-way (block × knowledge × sequence) repeated measures analysis of variance (ANOVA) was used to compare differences in RT between the blocks and groups. Separate pairwise comparisons were conducted on the priori contrasts of interest (block 1 vs. block 4 and block 4 vs. block 5) to determine any significant differences between the sequenced blocks and the random block. A three-way (block × knowledge × probability) ANOVA was used to compare differences in RT of stimuli with different probabilities in the two probabilistic groups. All repeated measures ANOVAs were performed in SAS with the MIXED procedure. Thus, the co-variance matrix structures were determined by the Akaike information criterion (AIC). A two-way (knowledge × sequence) ANOVA was employed to examine the effects of PK and sequence type on online, offline learning, and corrected offline learning. A two-way (knowledge × sequence) ANOVA was employed to examine the effects of PK and sequence type on the recall score. Given the violation of the normality assumption, the effects of PK and sequence type on the recognition score was examined by the Scheirer-Ray-Hare test. Tukey-Kramer *post hoc* tests were used to decompose any significant effects. Student’s *t*-tests/Wilcoxon tests were used to examine whether recall/recognition scores were different from the corresponding chance level for each group. The statistical significance level was set as *α* = 0.05.

## Results

**Figure 2:**
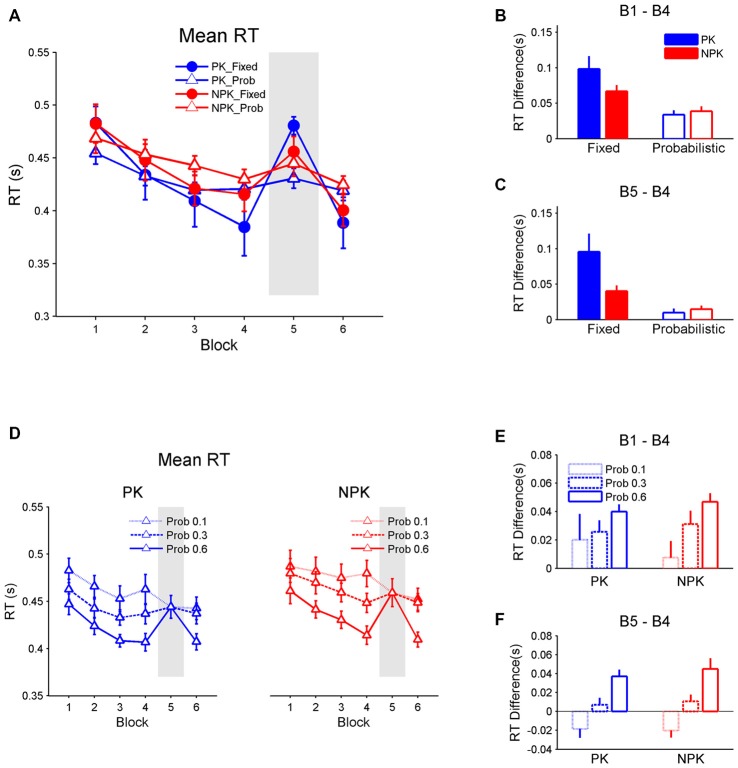
**(A)** Mean RT and SE bars across the six blocks for all four groups. **(B)** Difference between the RT in block 1 and block 4 to assess whether sequence learning occurred. **(C)** Difference between block 5 and block 4 to assess whether RT increases in block 5 when a random sequence is presented. **(D)** Mean RT and SE bars across the six blocks for only the probabilistic sequence in which the three transitional probabilities (Pro 0.1, Pro 0.3, and Pro 0.6) have been extracted and plotted separately. **(E)** Difference between RT in block 1 and block 4. **(F)** In block 5 and block 4 separated for the three transitional probabilities in the probabilistic sequence. PK, preliminary knowledge; NPK, no preliminary knowledge; RT, reaction time; SE, standard error.

Figure 2A shows the mean RT across the six blocks. The repeated measures ANOVA reveals a significant interaction between PK, sequence type, and block (*F*_(5,44)_ = 2.79, *p* < 0.05). *Post hoc* analyses with the Tukey-Kramer correction found that all four groups produced comparable RTs in all blocks (all *p* > 0.2). However, RT in two groups who performed the fixed sequences (i.e., PK_Fixed and NPK_Fixed) improved from blocks 1 to 4 and 6 (all *p* < 0.0001). In contrast, RT remained the same from block 1 to 4 in the other two probability sequence groups (i.e., PK_Prob and NPK_Prob, Figure 2B; all *p* > 0.1). Nevertheless, RT was faster in block 6 compared to block 1 in the NPK_Prob group (*p* < 0.01) and this improvement approached significance in the PK_Prob group (*p* = 0.09). In addition, when a random sequence was introduced in block 5, RT in the PK_Fixed and NPK_Fixed groups deteriorated (both *p* < 0.0001) while it remained the same between blocks 4 and 5 in the PK_Prob and NPK_Prob groups (both *p* = 1; Figure 2C).

The inferior learning in the probabilistic sequence (as expressed in no change in RT from block 1 to 4 and between blocks 4 and 5) is consistent with the hypothesis that probabilistic sequences are harder to learn compared to fixed sequences (Schvaneveldt and Gomez, 1998). However, given our hypothesis that participants typically acquire the sequence transitions of higher probabilities (Hunt and Aslin, 2001; Howard et al., 2004; Bornstein and Daw, 2012), the marginal learning effect on the probabilistic sequence likely resulted from the difference in RT among stimuli with different transitional probabilities (Figure 1C). Thus, we compared RTs between these stimuli (Figure 2D) in the probabilistic sequence. A three-way (block × knowledge × probability) repeated measures ANOVA found that PK does not significantly affect RT and there was a significant interaction between block and probability (*F*_(10,220)_ = 17.07, *p* < 0.0001). *Post hoc* analyses with the Tukey-Kramer correction revealed that RTs of stimuli with a transitional probability of 0.1 were comparable to that of stimuli with transitional probability of 0.3, while RTs of stimuli with transitional probability of 0.3 were slower than that of probability of 0.6 (*p* < 0.01). However, as learning progressed, RTs of stimuli with a transitional probability of 0.1 remained the same. In contrast, RTs improved from blocks 1 to 4 in stimuli with higher transitional probabilities 0.3 (*p* < 0.01) and 0.6 (*p* < 0.0001), suggesting learning of these higher transitional probabilities (Figure 2E). Additionally, introduction of a random sequence in block 5 did not impair RT of stimuli with transitional probabilities of 0.1 and 0.3, but RTs of stimuli with a transitional probability of 0.6 deteriorated in block 5 (*p* < 0.0001; Figure 2F). These results confirm that the participants learned stimulus transitions with higher probabilities, specifically 0.6 and perhaps 0.3.

**Figure 3:**
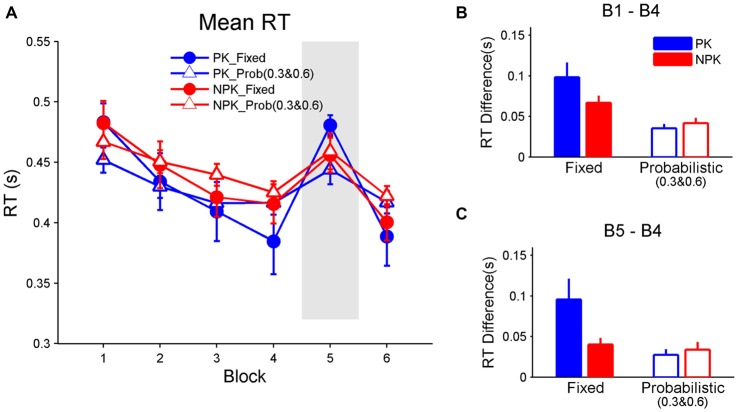
**Mean RT and SE bars to assess learning.** Only the RT of stimuli with transitional probabilities of 0.3 and 0.6 were extracted and are shown for the probabilistic sequences. **(A)** Mean RT across the six blocks. **(B)** Difference between the RT in block 1 and block 4 to assess whether sequence learning occurred. **(C)** Difference between block 5 and block 4 to assess whether RT increases in block 5 when a random sequence is presented. PK, preliminary knowledge; NPK, no preliminary knowledge; RT, reaction time; SE, standard error.

Since participants only learned higher transitional probabilities when stimuli followed a probabilistic pattern, we re-compared the learning effects among groups by using RT for stimuli with transitional probabilities 0.3 and 0.6 in PK_Prob and NPK_Prob groups. A repeated measures ANOVA revealed a significant interaction among the effects of block, PK, and sequence (*F*_(5,44)_ = 3.1, *p* < 0.05). Tukey-Kramer-corrected *post hoc* analyses suggest that all groups had comparable mean RTs across all blocks (Figure 3A). In addition, all groups demonstrated improved mean RT from block 1 to 4 (all *p* < 0.0001) and deteriorated mean RT from block 4 to 5 (all *p* < 0.005). However, contrast analyses showed that the PK_Fixed group had the greatest change in RT from block 1 to 4 compared to the NPK_Fixed (*p* < 0.05), PK_Prob (*p* < 0.0005), and NPK_Prob groups (*p* < 0.0005; Figure 3B), while the latter three groups exhibited the same change in RT. Similarly, the RT change from block 4 to 5 was greater in the PK_Fixed group compared to the other three groups (all *p* < 0.01) who had the same RT change (Figure 3C). These results suggest that the PK_Fixed group learned better than the other three groups.

**Figure 4:**
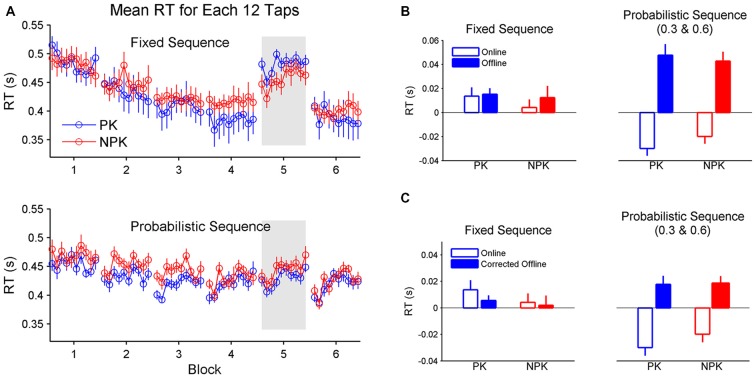
**(A)** Mean RT of each 12 taps to reflect online and offline learning. **(B)** Comparison of online and offline learning between the groups. **(C)** Comparison of online and corrected offline learning between the groups. Error bars represent standard errors. PK, preliminary knowledge; NPK, no preliminary knowledge; RT, reaction time.

Although participants learned either fixed or probabilistic sequences with or without PK of the sequence, learning across trials exhibited different patterns (Figure 4A). Specifically, learning of a fixed sequence exhibits decreased RT within blocks while learning of a probabilistic sequence exhibits reduced RT after rest without practice. A two-way (knowledge × sequence) ANOVA found a significant effect of sequence on offline learning (*F*_(1,44)_ = 8.84, *p* < 0.005). Particularly, the acquisition of the probabilistic sequence arises from greater offline learning compared to the acquisition of the fixed sequence (Figure 4B). Although sequence type was also found to significantly affect online learning (*F*_(1,44)_ = 18.72, *p* < 0.0001), it was shown that greater online learning was produced when a fixed sequence was learned (Figure 4B). Interestingly, when learning a probabilistic sequence, participants did not exhibit online learning. Instead, RT became slower within blocks. We further compared whether online or offline learning contributed more to the acquisition of a motor sequence. A two-way (knowledge × sequence) ANOVA on the RT difference between offline and online learning revealed a significant effect of sequence type (*F*_(1,44)_ = 15.27, *p* < 0.0005). Student’s *t*-tests found equal online and offline learning when a fixed sequence is performed (*p* = 0.59), while greater offline compared to online learning was found when a probabilistic sequence was performed (*p* < 0.0001).

We also analyzed the corrected offline learning. The same results were found compared to the original offline learning data (Figure 4C). A two-way (knowledge × sequence) ANOVA found a significant effect of sequence (*F*_(1,44)_ = 4.99, *p* < 0.05). Specifically, there was greater corrected offline learning in PK_Prob and NPK_Prob groups compared to PK_Fixed and NPK_Fixed groups. These results suggest that offline learning rather than reactive inhibition/fatigue underlies the offline improvement in RT.

**Figure 5:**
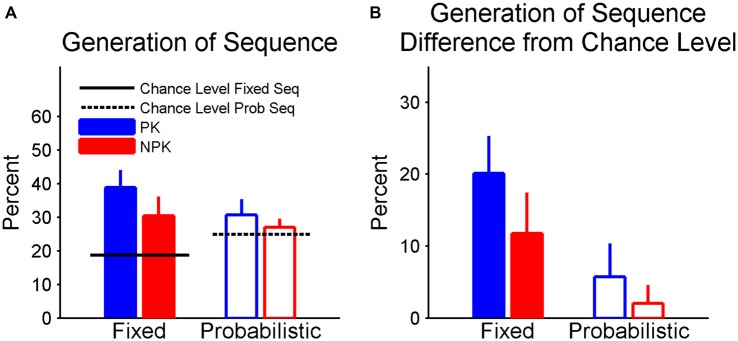
**(A)** Percentage of three-element chunks recalled in the posttest. **(B)** Corrected percentage according to chance level. Error bars represent standard errors. PK, preliminary knowledge; NPK, no preliminary knowledge.

In the posttest, we found that the recognition score did not differ from chance (i.e., 50%) in all four groups and there were no effects of sequence type and PK on the scores. Figure 5A shows the percentage of recalled three-element chunks. It is clear that participants in the fixed sequence groups had higher than chance recall, while recall was at chance in the two probabilistic sequence groups. The corrected percentage according to the chance level was shown in Figure 5B. A two-way ANOVA found a significant effect of sequence type (*F*_(1,44)_ = 6.75, *p* < 0.01). Specifically, recall of the fixed sequence was superior compared to that of probabilistic sequence. In addition, using Student’s *t*-tests with an adjusted *p* level of $\frac{\alpha }{4}$ = 0.0125 to control the familywise error rate for the four simultaneous *t*-tests, the recall in the PK_Fixed was significantly higher than chance level (*p* < 0.0001) The recall in the NPK_Fixed did not differ from chance (but approached significance, *p* = 0.0146), In contrast, recall in the two groups that performed the probabilistic sequence (i.e., PK_Prob and NPK_Prob) was not significantly different from the chance level (both *p* > 0.2).

## Discussion

In this study, we demonstrated that both fixed and probabilistic motor sequences can be learned quickly (i.e., in one training session). Further, this initial acquisition of a fixed sequence in the fast learning stage arises from both online and offline learning, while acquisition of a probabilistic sequence is driven predominantly by offline learning. Given that learning a probabilistic or fixed sequence requires greater procedural or declarative memory, respectively, our results suggest that a bias toward procedural or declarative memory modulates how a motor sequence is learned in the fast learning stage.

Offline learning, as a salient feature underlying motor sequence learning (Robertson et al., 2004a), can boost the memory of a newly acquired sequence 5–30 min after the initial acquisition (Albouy et al., 2006; Hotermans et al., 2006, 2008; Schmitz et al., 2009; Nettersheim et al., 2015) or consolidate the memory a few hours later without sleep (Robertson et al., 2004a; Brown and Robertson, 2007a,b) or after sleep (Walker et al., 2002; Robertson et al., 2004b; Censor et al., 2012; Nettersheim et al., 2015). Thus, offline learning has been widely considered to occur only after the initial acquisition of sequences that develops over the course of a single training session, referred to as fast learning (Honda et al., 1998; Karni et al., 1998; Walker et al., 2002; Dayan and Cohen, 2011; Censor et al., 2012). Unlike the widely-found offline learning that occurs following the fast learning stage, we observed offline learning that drives the fast acquisition of sequences within a first single training session. This result suggests that in addition to online learning (Cleeremans and McClelland, 1991; Bornstein and Daw, 2012), offline learning also contributes to rapid improvements in performance that allow sequences to be learned quickly in a single training session.

The concurrent effect of online and offline learning could be modulated by the involvement of declarative and procedural memory. It is widely accepted that both memory systems cooperate and compete during motor sequence learning (Meulemans et al., 1998; Brown and Robertson, 2007b). Remarkably, the presence of declarative memory inhibits offline learning of procedural memory and thus disruption of declarative memory induces offline improvement in procedural skills 4 h after the initial acquisition (Brown and Robertson, 2007a). In our study, similar effects of declarative and procedural memory were observed on offline learning in the fast learning stage. The recognition and recall tests were used to measure the engagement of declarative and procedural memory in the SRT task. Although the recognition test shows no differences in the amount of declarative knowledge acquired by participants regardless of the sequence type and PK (see details below), the recall scores reveal that, participants acquired less declarative knowledge of the probabilistic sequence. Notably, participants exhibited greater offline learning when performing probabilistic sequences, suggesting that the offline learning in the fast learning stage was strengthened when greater procedural memory and less declarative memory were required to learn the motor sequences. On the other hand, when greater declarative memory was involved in learning fixed sequences, as indicated by higher recall scores, reduced offline and greater online learning were observed. This inverse relationship between online and offline learning confirms the inhibition effect of declarative memory on offline learning. More importantly, our finding extends our understanding of the competition between multiple memory systems. That is, unlike previous studies that demonstrated this competition after skills are acquired (Poldrack et al., 2001; Foerde et al., 2006; Brown and Robertson, 2007b), we demonstrated that the competition begins as soon as learning starts and that declarative and procedural memory may be identified by their distinct behavioral expressions.

The offline learning observed within a single training session (i.e., the fast learning stage) is associated with procedural memory as is offline learning that takes place hours after the initial acquisition and is responsible for memory consolidation. However, it remains unclear whether this offline learning that allows fast initial acquisition of a motor sequence is related to offline learning that consolidates the memory of a newly acquired sequence. It is possible that offline learning that drives the fast acquisition is a precursor of the later occurring memory consolidation, or they may be the same process. To elucidate their relationship, further systematic investigations are needed.

A debate within the offline learning literature is whether offline improvement in performance after rest, referred to as reminiscence (Eysenck and Frith, 1977), results from fatigue or reactive inhibition (Rickard et al., 2008; Brawn et al., 2010) or an active learning mechanism (Eysenck and Frith, 1977; Robertson et al., 2004a). It has been suggested that offline learning and reactive inhibition/fatigue are usually combined to lead to reminiscence (Eysenck, 1965), thus making it difficult to determine if reactive inhibition/fatigue is a potential cause of reminiscence. However, observations from our data favor offline learning to reactive inhibition/fatigue as the primary mechanism underlying offline improvement in RT or reminiscence observed in the SRT task. Specifically, with the same amount of practice, only participants who performed the probabilistic sequence slowed down their RT, while such “fatigue” was not observed when participants performed a fixed sequence. In addition, if fatigue appeared as soon as participants in the probabilistic sequence groups started to perform the task, it would be unlikely that their learning would arise quickly (i.e., over four learning blocks) and to a comparable level as the participants in the fixed sequence groups who did not exhibit fatigue. Moreover, according to Eysenck and Frith (1977), reminiscence is task-specific. For example, reminiscence that results from reactive inhibition or fatigue usually occurs in a task that does not involve learning, where performance on the task is already perfect when an individual starts to perform the task. In contrast, reminiscence that arises from offline learning usually takes place in a learning task. Obviously, the SRT task involves sequence learning and our data demonstrated that participants learned the sequence. Further evidence supporting offline learning rather than reactive inhibition or fatigue comes from the observation on corrected offline learning. In the probabilistic sequence groups, performance after the short break is superior to the best performance level before the break. Therefore, without fully excluding the effect of reactive inhibition/fatigue, our results favor the statement that the offline improvement in RT is driven by offline learning rather than reactive inhibition or fatigue. Meanwhile, we suggest that it is necessary to systematically examine the reactive inhibition or fatigue effects in future sequence learning studies.

Although it appears that offline learning rather than reactive inhibition or fatigue is the primary mechanism underlying the offline improvement in RT, the cause of increased RT when learning a probabilistic sequence is unclear. One likely reason is the interference of stimuli transitions with a probability of 0.1. It has been found that adults learned a sequence by iteratively updating the internal model of the motor sequence (Cleeremans and McClelland, 1991; Bornstein and Daw, 2012, 2013; Verstynen et al., 2012) and our data provide consistent evidence that participants acquired the stimulus transitions with probabilities of 0.3 and 0.6. However, the introduction of stimulus transition governed by a probability of 0.1 may mislead the updating of the internal model (i.e., transitional probability matrix) and thus impair RT when the probabilistic sequence was performed.

In addition to the primary findings on online and offline learning, our results provide insights into the learning of probabilistic sequences. Sequence structure plays a critical role in motor sequence learning (Curran and Keele, 1993; Jiménez et al., 1996; Bennett et al., 2007; Song et al., 2007). To date, a variety of probabilistic sequences have been used in the SRT task, but only a few studies have employed probabilistic sequences that represent the stochastically related events of daily life, such as sequences produced by a finite state grammar (Jiménez et al., 1996) or a Markov chain. We found that participants acquired stimulus transitions with higher probabilities of 0.3 and 0.6 and the learning of these higher stimulus transitions was comparable to that of the fixed sequence. Moreover, the facilitating effect of PK of a sequence depends on the sequence structure, which is consistent with previous studies (Jiménez et al., 1996; Stefaniak et al., 2008). Specifically, PK only facilitates the learning of a simple sequence, such a fixed sequence (Curran and Keele, 1993; Frensch and Miner, 1994; Curran, 1997; Destrebecqz, 2004; Stefaniak et al., 2008) and not a sequence with a complex structure.

Finally, one caveat worthy of further study is the measurement of the amount of declarative knowledge. Both recognition and recall tests are most widely used to examine procedural learning in the SRT task (Shanks and Johnstone, 1999; Wilkinson and Shanks, 2004; Destrebecqz and Peigneux, 2005). In particular, these tests examine whether participants can explicitly recollect the acquired sequence knowledge. However, results from the recognition tests are equivocal in the literature (Perruchet and Amorim, 1992; Willingham et al., 1993; Reed and Johnson, 1994; Shanks and Johnstone, 1999). Similarly in our study, unlike the recall tests demonstrating the common finding that probabilistic sequence learning favors more procedural memory (Jiménez et al., 1996; Song et al., 2007), the recognition tests reveals no difference in the amount of acquired declarative knowledge despite the sequence type and PK. In addition, the recognition scores in all four groups were not greater than chance. Given that in the recognition test, participants were presented with sequence segments and were asked to determine whether these segments are from the sequence they learned or a new sequence they did not see in the SRT task, it is hard to know whether the chance-level score was due to the participant’s inability to explicitly recollect sequence knowledge or that the participant did not learn some segments of the sequence. These two possibilities that may simultaneously account for the chance-level recognition must be addressed by other tests in future studies. Moreover, in our study, only four correct sequence segments were given to participants, while there were more than 10 segments within the learned sequence, the chance-level recognition score was caused possibly because some participants may learn segments other than the four displayed in the recognition test.

In summary, we found that concurrent online and offline learning allows motor sequences to be acquired quickly in the fast learning stage and can be identified by their manifestations in the progressive changes in RT. Remarkably, online and offline learning can be mediated by the declarative and procedural memory that are required to learn motor sequences. In addition, the modulation of online and offline learning may reflect the competition between both memory systems during motor sequence learning that begins in the fast learning stage. How the offline learning that drives the initial acquisition of sequences is related to the offline learning that is responsible for memory consolidation occurring hours after the initial acquisition remains to be investigated.

## Author Contributions

YD, SP, IS, and JEC designed the experiment. IS performed the experiment. YD and SP analyzed the data and prepared all tables and figures. YD, SP, and JEC wrote the manuscript. All authors reviewed the manuscript.

## Conflict of Interest Statement

The authors declare that the research was conducted in the absence of any commercial or financial relationships that could be construed as a potential conflict of interest.
